# Examining media reports of pediatric unintentional firearm injury deaths for prevention messaging concerning secured storage of firearms: U.S., 2021–2022

**DOI:** 10.1186/s40621-024-00485-6

**Published:** 2024-02-13

**Authors:** Bart Hammig, Abigail Bordelon, Corinne Chandler

**Affiliations:** Public Health Program, University of Arkansas, Fayetteville, AR USA

**Keywords:** Firearms, Pediatric, Media, Safe storage, Unintentional

## Abstract

**Background:**

Media outlets that report on firearm injuries and deaths may provide an important role in emphasizing safe storage practices, particularly when unintentional firearm injuries and deaths occur among young children. There has been a scarcity of research on aspects of media reports on injuries, particularly concerning prevention messaging. The objective of the present study was to examine prevention messaging on the safe storage of firearms among media outlets when reporting on unintentional firearm injury deaths among children aged 0–11.

**Methods:**

The Gun Violence Archive collects information from media sources regarding firearm injuries and deaths. We analyzed data from 2021 to 2022 to analyze prevention messaging incorporated into the media reports. We then examined if media reporting of events that occurred in States with child access prevention (CAP) laws had a higher likelihood of including prevention messaging compared to those events occurring in States without CAP laws.

**Results:**

There were 223 deaths reported that were attributed to unintentional firearm discharge among children aged 0–11. Prevention messages were delivered in 61 of the 223 incidents. Specific messages which included the word “lock” when referring to firearm storage were included in 21.9% of all incidents. An analysis examining State CAP laws and the presence of any prevention message per event did not yield any discernable relationship.

**Conclusion:**

Inclusion of prevention messaging stating that firearms should be stored locked and unloaded when reporting on unintentional firearm injury deaths among children is lacking. When specific prevention messaging was included, the source was often law enforcement. Public health officials may play an important role in educating the journalistic and law enforcement communities about the inclusion of safe storage messages when reporting on firearm deaths.

## Introduction

Firearm injury is a major public health problem in the United States, accounting for more than 45,000 deaths annually (Centers for Disease Control and Prevention 2023a; Davis et al. 2023). Since 2017, firearms surpassed motor vehicle crashes as the leading cause of death for children, youth and young adults aged 0–24 (Centers for Disease Control and Prevention 2023b; Lee et al. 2022). While the rise in firearm deaths among adolescents and young adults is largely due to suicide and homicide, deaths among younger age groups due to unintentional discharge of a firearm remain an important public health issue. While homicides account for the leading cause of firearm deaths among pre-adolescent children, unintentional deaths due to firearms are more common in these age groups when compared to older children and adolescents (Hemenway and Solnick 2015; Vaishnav et al. 2023). The number of children killed by unintentional firearm discharges are largely misclassified as homicides in vital statistics data, resulting in a pronounced undercount of cases (Hemenway and Solnick 2015). As such, trends in the number of pediatric unintentional deaths are difficult to quantify, but likely result in approximately 110 deaths per year. Compared to other high-income countries, the United States is unparalleled with regard to pediatric firearm deaths, accounting for more than 90% of all deaths globally (Grinshteyn and Hemenway 2019). Moreover, most firearm-related deaths occur in the child’s home, and approximately 30 million children in the U.S. live in a household with a firearm (Fowler et al. 2017; Miller and Azrael 2022).

The number of guns sold in the U.S. has doubled over the past 20 years, with sharp increase in sales since 2019. During the COVID 19 pandemic, one fifth of U.S. households purchased guns, with nearly 60 million guns purchased between 2020 and 2022 (Walsh 2023). A rise in gun sales and the resultant increase in the number of guns in homes presents a public health concern, as the most common antecedent event associated with unintentional childhood deaths caused by firearms is a child playing with a gun that was not securely stored in the home (Hemenway and Solnick 2015; Lee et al. 2022; Vaishnav et al. 2023). To prevent unintentional firearm injury deaths among the young pediatric population, gun owners are recommended to adhere to secure firearm storage practices and/or regulations. The Center’s for Disease Control and Prevention (CDC) has emphasized that “putting a firearm out of sight or out of reach is not safe storage and not enough to prevent use by children or unauthorized adults The American Academy of Pediatrics (AAP) and The National Shooting Sports Foundations ChildSafe project both take similar harm reduction approaches to the storing of firearms(American Academy of Pediatrics 2023; The National Shooting Sports Association 2023). Specifically, the AAP recommends in households that contain firearms, the firearm should be stored locked and unloaded, with the ammunition also locked and stored separately from the firearm (American Academy of Pediatrics 2023). Some states have implemented laws to regulate firearm storage within homes in an effort to make firearms less accessible to children and adolescents. Child Access Prevention, or CAP laws, aim to reduce childhood firearm deaths by allowing prosecution of adults who allow children to have unsupervised access to a firearm (The Rand Corporation 2023). The extent of child access prevention (CAP) laws vary by state and may influence the extent of pediatric and adolescent firearm deaths and injuries in each state, though evidence is mixed (Hamilton et al. 2018; Hepburn et al. 2006; Miller et al. 2022). Evidence does suggest that states with strong CAP laws (those that impose criminal liability charges for adults when a child obtains a firearm in their possession due to improper storage or negligence) may be an effective measure to reduce unintentional firearm injuries (Hamilton et al. 2018). However, with regard to behavior, findings also suggest that CAP laws have not resulted in a change in storage practices among gun owners who live in states with CAP laws. Research has revealed that 55% of gun owners did not know if they lived in a state with CAP laws. Moreover, gun owners who resided in states with CAP laws were no more likely to store their firearms securely that those who resided in states without CAP laws. (Miller et al. 2022).

Parents often perceive that children will not handle a firearm if found and therefore underestimate the risk (Baxley and Miller 2006; Connor and Wesolowski 2003). The most recent data indicate that approximately 50% of all gun owners reported storing their guns in some sort of safe storage practice that included a lock, such as a gun safe or trigger lock (Crifasi et al. 2018). In addition to CAP laws, other factors have been examined that may influence the utilization of safe storage practices. These include gun safety training courses that promote safe storage, and anticipatory guidance by pediatricians, both of which have been shown to improve safe storage practices (Crifasi et al. 2018; Lee et al. 2022). Furthermore, the source of the information about safe storage practices has been shown to be of pertinent concern to gun owners, with law enforcement, hunting organizations, military personnel and the National Rifle Association perceived to be the most credible sources concerning safe storage practices (Crifasi et al. 2018). While safe storage recommendations may be delivered during gun safety training courses or anticipatory guidance from pediatricians, it remains that almost half of all gun owners do not safely store their firearms and 39% of gun owners do not undergo safety training (Rowhani-Rahbar et al. 2018). Moreover, pediatricians provide firearms safety counseling in only 1–5% of all well child visits, and the majority do not inquire about firearm safety with their patients (Hoops and Crifasi 2019). To compound the issue, states are increasingly eliminating permits and/or training to legally carry a firearm, and only 9 states require gun purchasers to have firearm safety training.

News media outlets that report on firearm injuries and deaths may provide an important role in emphasizing safe storage practices, particularly when unintentional firearm injuries and deaths occur among young children. A tragic event such as the unintentional death of a child from a firearm can serve as a cue to action for others in the community, and it may provide an opportunity for the news media to inform the public about specific prevention measures that may reduce the risk of a future fatality (Clegg Smith et al. 2007). There has been a scarcity of research on aspects of news media reports on injuries during the past 2 decades, particularly concerning the framing of prevention messages. The research that has been conducted has indicated that news media outlets are deficient in communicating injury prevention information (Clegg Smith et al. 2007; John and Kool 2017; Ozegovic and Voaklander 2011; Voight et al. 1998). The most recent study found that the majority of news media reports on childhood injury did not contain prevention messaging, and only 20% included clear prevention messages to the public (John and Kool 2017). However, the limited amount of research has shown that prevention messaging through news media outlets have led to changes in injury prevention behavior. For example, Bergman and colleagues utilized the news media to advocate for, and subsequently increase, bicycle helmet used in Seattle (Bergman et al. 1990). An increase in suicides through suicide contagion, for example, has been shown to be influenced by the how the story was framed in news media reports about the death (Bohanna and Wang 2012; Niederkrotenthaler et al. 2020, 2010). This has led to the development of journalistic reporting guidelines as an effective way to prevent suicide contagion by incorporating reporting methods that avoid sensationalism and details on the method of suicide, while at the same time including prevention messaging such as access to help lines (Bohanna and Wang 2012; Niederkrotenthaler et al. 2020).

Successful prevention of firearm injuries and deaths will likely need to employ multifaceted intervention approaches, akin to tobacco prevention in the U.S. (Cummings and Proctor 2014). Research has indicated that the news media can influence public health behaviors, and news media outlets who report on firearm-related morbidity and mortality have a platform from which they can educate and encourage safe storage practices. Using the Gun Violence Archive (GVA), we sought to examine the use of prevention messaging among news media outlets when reporting on unintentional firearm injury deaths among children aged 0–11. Moreover, we sought to discern whether news media reporting incorporated guidelines set forth by the CDC and AAP when reporting on safe storage of firearms. Lastly, we assessed CAP laws by state and their association with prevention messaging from news media outlets.

## Methods

### Data and measures

The GVA (The Gun Violence Archive 2023) is a web-based research and data collection repository that collects information from over 7500 print and broadcast news media sources regarding firearm incidents, including, but not limited to “accidental” deaths, homicide, suicide, and non-fatal injuries. Established in 2013, the GVA utilizes source inclusive of law enforcement, news media (print and broadcast), data aggregates, and government sources on a daily basis (The Gun Violence Archive 2023). While the GVA data are constantly updated per event, we used data from the most recent full calendar years 2021–2022. We examined reports for “accidental deaths (Children Ages 0–11)” to investigate whether or not a prevention message was included in at least one news media report associated with each event. Because many events included several news media reports, as the story evolved, we anecdotally noticed that the first reports typically mentioned a death with little context, and the latter news media accounts focused on criminal prosecution. In general, prevention messages, if mentioned, occurred in early news media reports and after specifics of the event were known. Therefore, we coded prevention messages using the denominator of each incident as opposed to total number of news media stories. We developed two definitions of a “prevention message.” We manually reviewed all news media accounts surrounding each incident. Where possible, transcripts were read to decipher the news media report. If a transcript was not available, but there was a multimedia clip (e.g., local TV news story), we viewed or listened to the news media clip associated with incident. First, a broad definition was employed in which we coded “yes” for a prevention message if the news media report mentioned keeping guns stored away from children. Examples included “Put it up in a secure area, if you think it’s a secure area, if the child sees it they’re going to try and get it because they’re curious about a handgun” and “So make sure when you’re securing it, don’t put it anywhere the child can see it, don’t even let the child see you putting wherever you store at it” and “The Milwaukee Police Department would like to remind the public to secure and keep firearms out of the reach of children.” We also developed a more specific prevention message definition which coincides with recommendations from the American Academy of Pediatrics and prior research in which the news media report included the word “lock” or “locked” with respect to firearms (Grossman et al. 2005; Schaechter 2022). Examples of news media reports that were coded as meeting this more specific definition included.” Children should never have access to a gun and that’s why it is imperative to secure your firearm in your home with a gunlock or inside a gun safe,” and “When we have weapons in our homes we need to make sure that we have a gun lock and keep these things out of the sight of our children.” While the recommendation from AAP is to keep firearms locked and unloaded, with ammunition locked separately, and this coincides with Everytown for Gun Safety motto of “unload, lock, and separate (Everytown for Gun Safety 2022),” we noted only one news media report that was this inclusive. Therefore, our more specific definition included the word “lock” or “locked” in the context of the firearm being secured.

### Case selection

Similar to a prior study using the GVA, we first read a sample of 15 gun-related fatalities with a corresponding news media report count of 44 news reports in order to develop a classification system for potential variables of interest (Hemenway et al. 2022). We then created a spreadsheet with noted variables and coded all fatal incidents and respective news media reports for 2021–2022. It should be noted that none of the incidents in the GVA during the study timeframe included multiple victims with regards to death by firearm; hence, each incident represents a single victim. Each incident ranged from 1 to 7 news media reports. Given that news media reports tended to change in purpose and scope as the incident was followed over time, data were analyzed at the incident level and not per news media report. Therefore, if any of the news media reports associated with the incident contained a prevention message, it was coded as such. We excluded a total of 42 incidents from our analyses. These included 17 cases where incident characteristics did not relate to safe storage practices (e.g., victim killed by a stray bullet from an unknown shooter), 12 cases where there was insufficient information about the incident, and 13 cases where the victim was older than 11 years of age.

### Coding and classification of variables

Variables included in our data set were date of incident, state, number killed, number injured, age of victim, gender of victim, number of children involved in the shooting, whether or not a caregiver was present, inclusion of a prevention message in the news media report, whether or not the message was specific or general, the messenger who deliver the message (law enforcement, journalist, other), age of shooter if not self-inflicted, antecedent to shooting incident (playing with gun or other) and type of firearm. We also coded each state based on CAP Laws, coded as either no law or having a CAP law (Giffords Law Center to Prevent Gun Violence 2023). Since all analyses were based on the incident, in the event that a news media from one state was reporting on a death in another State, the CAP law was categorized based on the state where the incident occurred and not from where the news media originated.

### Analyses

Descriptive epidemiological analyses were conducted to examine the reporting characteristics of unintentional firearm deaths by news media outlets. We then utilized a Pearson Chi-square test to examine if news media reporting of events that occurred in states with CAP laws had a higher likelihood of prevention messaging compared to those events occurring in states without CAP laws. Inter-rater reliability for prevention message coding was conducted for those cases originally identified as containing a prevention message. Cases were divided into three portions, with one researcher then re-coding 1/3 of the cases already coded by another researcher. A total of 6 cases originally coded as containing a prevention message were either reversed and subsequently coded as not containing a prevention message or changed from specific to not specific, for a percent agreement of 90.0%. This study used publicly available data and was deemed exempt by the Institutional Review Board.

## Results

### Descriptive and demographic findings

The GVA reported on 265 unintentional deaths due to firearms among children aged 0–11 between 2021 and 2022. An average of 3 news stories were reported per incident, with a range of 1–7 news stories. The final case count resulted in 223 cases of unintentional deaths in which storage of a firearm could have been a contributing factor to the incident.

The majority of decedents were male (75.0%) and aged 2–6 years. Most of the incidents occurred in a home or residence, with 4% occurring elsewhere (e.g., child found gun in automobile). Antecedent events included ‘playing’ with a firearm, representing 74.9% of the events, with 53.4% having been unintentional self-inflicted gunshots. Among the events in which the injury was not self-inflicted, the age of the shooter ranged from 2 to 16 + , with the majority being 16 years of age or older. Sixty-three percent of the decedents were aged 2 to 6 years. Among children who died by a self-inflicted gunshot (n = 119), 79.8% were between the ages of 2 and 6 years. See Tables 1 and 2.

**Table 1:** Characteristics of unintentional firearm deaths among children aged birth to 11 years, gun violence archive 2021–2022 (*N* = 223)

	Number	%
Sex		
Male	167	75.0
Female	49	22.0
Unk/not reported	7	3.0
Age of victim		
0–1	15	6.7
2–6	141	63.2
7–11	66	29.6
Unk/not reported	1	0.5
Location of incident		
Residence/home	204	91.5
Other	9	4.0
Unk/not reported	10	4.5
Antecedent event		
Playing with firearm	167	74.9
Other	17	7.6
Unk/not reported	39	17.5
Type of firearm		
Handgun	85	38.1
Other	12	5.4
Unk/not reported	126	56.5
Self-inflicted		
No	92	41.3
Yes	119	53.4
Unk	12	5.4
Age of shooter if not self-inflicted (*n* = 92)		
0–1	0	0
2–5	10	4.5
6–10	18	8.1
11–15	7	7.0
16 +	46	20.6
Unk/not reported	11	5.4
Caregiver present		
No	27	12.1
Yes	138	61.9
Unk/not reported	58	26.0

Source:(*The Gun Violence Archive*, 2023)

**Table 2:** Prevention messages presented in media reports of unintentional firearm deaths among children aged birth to 11 years, gun violence archive 2021–2022 (*N* = 223)

	Number	%
Prevention message		
No	162	72.7
Yes	61	27.4
Prevention messenger (*n* = 61)		
Other	11	18.0
Police/Law enforcement	50	82.0
Specific prevention message (*n* = 61)		
No	12	19.7
Yes	49	80.3

*Source* The Gun Violence Archive (2023)

### CAP Laws and prevention messaging

An analysis examining state CAP laws and the presence of any prevention message per event did not yield any discernable relationship. Among those states without CAP laws (*n* = 33), 25.9% of news media stories included a prevention message, and in those states with CAP laws (*n* = 17), 29.6% of news media stories included a prevention message (PChisq = 0.35, *p* = 0.55). See Table 3.

**Table 3:** Child Access Prevention Laws by State through 2022, and Association Between States with CAP Laws and States Without with Regard to Prevention Messaging in Media Reports of Unintentional Firearm Deaths Among Children Aged 0–11

States with CAP Law (88 incidents reported)	States Without CAP law (135 incidents reported)
California	Alabama
Colorado	Alaska
Connecticut	Arizona
Delaware	Arkansas
Florida	Georgia
Hawaii	Idaho
Illinois	Indiana
Iowa	Kansas
Maine	Kentucky
Maryland	Louisiana
Massachusetts	Mississippi
Michigan	Missouri
Minnesota	Montana
Nevada	Nebraska
New Hampshire	North Dakota
New Jersey	Ohio
New York	Oklahoma
North Carolina	Pennsylvania
Oregon	South Carolina
Rhode Island	South Dakota
Texas	Tennessee
Vermont	Utah
Virginia	West Virginia
Washington	Wyoming
Wisconsin	New Mexico (not effective until June 2023)

Pearson Chi^2^ = .3512, *p* = .553

Adapted from the Giffords Law Center to Prevent Gun Violence https://giffords.org/lawcenter/gun-laws/policy-areas/child-consumer-safety/child-access-prevention-and-safe-storage/

Prevention messages were delivered in 61 of the 223 incidents. Specific messages which included the word “lock” or “locked” when referring to firearm storage were included in 21.9% of all incidents. Among the events in which a news media report included a prevention message, 80.3% included a specific prevention message, while 19.7% included a general prevention message. When prevention messaging was delivered, 82.0% of the time it was communicated by law enforcement. When we examined the 61 prevention messages by crosstab of who the messenger was by specific vs. non-specific prevention message, we found that among the 50 law enforcement officials that delivered a prevention message, 38 delivered a specific prevention message, and 11 delivered a non-specific prevention message. Among the “other” category, which included politicians, journalists, community leaders, etc., all 11 delivered a specific prevention message, and none delivered a non-specific prevention message. Lastly, while 56.5% of the firearms used in the event were not reported or unknown by type, 38.1% of the events were caused by a handgun. A caregiver was deemed present in 61.9% of the events, with 26.0% either unknown or not reported.

## Discussion

Our core areas of focus for this study were to examine the news media coverage of unintentional deaths resulting from firearms among children 0–11 for the years 2021 and 2022, and to examine what, if any, association between a state’s CAP laws and number of unintentional shooting deaths existed. We used the GVA which provides news articles about firearms incidents across the United States.

Coinciding with prior research on fatal and non-fatal injuries, we found a lack of prevention messaging attached to the news media reports surrounding unintentional firearm injury deaths among children (Clegg Smith et al. 2007; John and Kool 2017; Ozegovic and Voaklander 2011; Voight et al. 1998). Only 49 of the 223 events reported on by the news media included a specific prevention message. Given that the majority of deaths had follow-up news media coverage, an opportunity to frame prevention messages on safe storage was lost. Many of the articles we examined included a quote from law enforcement or referenced a news media release from a law enforcement agency, therefore, a logical step that could be taken to provide more comprehensive reporting of unintentional shootings would be to train both journalists and law enforcement officials on how to consistently deliver safe storage messaging when reporting on firearm injuries and deaths. It is routine for law enforcement agencies to release a statement when a person dies as a result of firearm violence, and incorporating a prevention message could become standard protocol. Additionally, law enforcement officers could be trained to communicate resources that are available in the community to help prevent firearm violence. For example, some municipal law enforcement agencies offer trigger locks and other safety devices that community members can get free of cost (The National Shooting Sports Association 2023). Moreover, the extent to which law enforcement officials utilize programs such as Project ChildSafe, which provide law enforcement agencies with free gun locks to disperse to the public is not known, but could be an avenue to engage the public in secure storage efforts (The National Shooting Sports Association 2023).

Law enforcement education is crucial, but ultimately it is largely journalists who are proliferating information about unintentional shootings to the public. A restructuring of how the news media reports on unintentional shootings in the pediatric population will not be a panacea, but may have a modest impact and is arguably a quicker step to be taken than reforming legislation. In an effort to increase prevention messaging, journalists could be provided guidance by public health entities on the types of questions that should be asked, and of whom, when reporting on firearm injuries and deaths. Similar to the approach taken for reporting on suicides and mass shootings in an effort to prevent a contagion effect, (Towers et al. 2015) guidelines for effective prevention messaging could be developed for inclusion of more specific messaging concerning the safe storage of firearms (Towers et al. 2015). Providing guidance and reasoning to journalists, news media providers, and law enforcement agencies to not refer to unintentional shootings as “accidental” is another step public health officials could embark upon. An overarching theme in the news media reports taken from the GVA was that the vast majority of shootings were deemed “accidental.” In fact, the GVA itself categorizes these unintentional shootings as “accidental shootings.” While terminology surrounding the use of the term “accident” has a complicated history in the injury field (Girasek 1999; Knechel 2015), we found that the context in which the term was often used connoted an injury that was not preventable (e.g., “this was just a tragic accident”). Future research on use of the term “accident” versus “unintentional” (which conveys a preventable incident) may consider the source, type of injury, and context in which the term “accident” is used.

We did not find a relationship between the use of prevention messaging in news media reports of events that occurred in states with CAP laws compared to states without CAP laws. While reasons for our findings are unclear, it may be that journalists are unware of CAP laws in their respective states, akin to findings that gun owners are often unaware of CAP laws (Miller et al. 2022). In states that have strong CAP laws, reminding the public through news media reports that CAP laws are in place may serve as additional motivation for gun owners to store firearms appropriately, or may provide an avenue to educate the public about CAP laws.

The juxtaposition of journalism and public health is complex. Public health has often appealed to the news media to convey important health information and to broadcast public health messages, especially during public health crises (Klemm et al. 2019). While a great deal of research has been produced to examine the news media’s role during outbreaks, such as the COVID 19 pandemic and Ebola epidemics (Anwar et al. 2020; Thomas and Senkpeni 2020); with the exception of suicide reporting guidelines(Niederkrotenthaler et al. 2010), there is a paucity of research or recommendations concerning individual-level events, such as firearm deaths or motor vehicle crashes, that may have a broader societal reach (De Ceunynck et al. 2015). Journalists who specialize in health and medical matters are likely better trained to report on these issues, especially if they affect large populations; however, public health events that affect few individuals may be more likely to be reported locally and by generalists in the field (De Ceunynck et al. 2015). This may in turn make the uptake of safe storage recommendations more difficult to invoke.

### Utility of the GVA and limitations

News media provided greater context than other forms of surveillance data with regard to antecedent events, location of the incident, whether or not the shooting was self-inflicted, (and if not, the age of the shooter), and location of the shooting. All of these provided more depth to the circumstances surrounding the incident which can lend itself to future prevention efforts. The GVA has been found to have good sensitivity and very high predictive value positive. In particular, fatal shootings and incidents involving children were found to have a high capture rate when compared to police data. However, those findings were based on 4 urban areas; therefore, results may differ for other geographic areas (Gobaud et al. 2023). The findings of this report are subject to at least 6 limitations. First, there was little to no data provided on the race or ethnicity of the victim. Second, the type of firearm used was missing in many cases, though based on sales data and the age of the victims, it is likely most were handguns. Third, since the GVA is not 100% complete in the reporting of unintentional firearm injury deaths, we could not establish a true mortality rate. Fourth, the GVA capture rate may be impacted by external influences on news media coverage (Gobaud et al. 2023). Factors such as politics, advocacy, and news media goals may increase or decrease reporting in shooting events. As indicated, since we analyzed the data at the incident level, our findings do not reflect the overall prevalence of prevention messaging per news media report. Fifth, the GVA utilizes a variety of sources and news media outlets to establish the database; however, it is unknown if the GVA relies on one news media type more than another. However, both print and broadcast news media were found when reviewing cases herein. Finally, circumstances for how the child or shooter retrieved the gun were not included in all cases. Those news media reports that did include from where the child retrieved the gun indicated that it was accessible and the child found and “played” with the gun. None of the news media reports indicated that a child had retrieved a firearm from a locked safe or trigger lock prior to the incident.

## Conclusions

In conclusion, the inclusion of prevention messaging stating that firearms should be stored locked and unloaded when reporting on unintentional firearm injury deaths among children is lacking. When specific prevention messaging was included, it usually came from a law enforcement officer. Moreover, state CAP laws did not appear to have an impact on news media messaging when reporting on shooting events. While it is unclear whether or not inclusion of such messaging by news media would result in an impactful change in storage practices, it may be an important component of a broader campaign to prevent unintentional firearm injury deaths among children. Future research is needed to examine the influence of prevention messaging on firearm storage behaviors, and whether this influence may vary based on demographic characteristics of the target population.

## Data Availability

All data are publicly available at https://www.gunviolencearchive.org/.
